# Prognostic value of ^18^F-FDG PET/CT radiomic model based on primary tumor in patients with non-small cell lung cancer: A large single-center cohort study

**DOI:** 10.3389/fonc.2022.1047905

**Published:** 2022-11-17

**Authors:** Jihui Li, Bin Zhang, Shushan Ge, Shengming Deng, Chunhong Hu, Shibiao Sang

**Affiliations:** ^1^ Department of Nuclear Medicine, The First Affiliated Hospital of Soochow University, Suzhou, China; ^2^ State Key Laboratory of Radiation Medicine and Protection, Soochow University, Suzhou, China; ^3^ Department of Radiology, The First Affiliated Hospital of Soochow University, Suzhou, China

**Keywords:** NSCLC, carcinoma, radiomics, PET/CT, prognosis

## Abstract

**Objectives:**

In the present study, we aimed to determine the prognostic value of the ^18^F-FDG PET/CT-based radiomics model when predicting progression-free survival (PFS) and overall survival (OS) in patients with non-small cell lung cancer (NSCLC).

**Methods:**

A total of 368 NSCLC patients who underwent ^18^F-FDG PET/CT before treatment were randomly assigned to the training (n = 257) and validation (n = 111) cohorts. Radiomics signatures from PET and CT images were obtained using LIFEx software, and then clinical and complex models were constructed and validated by selecting optimal parameters based on PFS and OS to construct radiomics signatures.

**Results:**

In the training cohort, the C-index of the clinical model for predicting PFS and OS in NSCLC patients was 0.748 and 0.834, respectively, and the AUC values ​​were 0.758 and 0.846, respectively. The C-index of the complex model for predicting PFS and OS was 0.775 and 0.881, respectively, and the AUC values ​​were 0.780 and 0.891, respectively. The C-index of the clinical model for predicting PFS and OS in the validation group was 0.729 and 0.832, respectively, and the AUC values ​​were 0.776 and 0.850, respectively. The C-index of the complex model for predicting PFS and OS was 0.755 and 0.867, respectively, and the AUC values ​​were 0.791 and 0.874, respectively. Moreover, decision curve analysis showed that the complex model had a higher net benefit than the clinical model.

**Conclusions:**

^18^F-FDG PET/CT radiomics before treatment could predict PFS and OS in NSCLC patients, and the predictive power was higher when combined with clinical factors.

## Introduction

Lung cancer is one of the most predominant malignancies worldwide, with a high incidence and mortality rate. Non-small cell lung cancer (NSCLC) is the most common pathological type of lung cancer, accounting for 85% of all lung cancer cases (1). Lymph node metastases or distant metastases often occur before diagnosis due to the lack of early and specific clinical signs, significantly affecting treatment and prognosis. The 5-year survival rate for lung cancer is less than 20% (2), while the availability of new drugs or treatments has improved survival rates for lung cancer patients. Even with the same treatment, the patients reflect a vast difference. Identifying the risk of recurrence at the time of diagnosis allows the patient’s treatment to be adjusted, thus enhancing the patient’s survival.

Preoperative conventional imaging methods are essential for determining TNM staging, which is traditionally used to assess treatment response or survival. However, it has some limitations, which produce a significant variation in survival durations for patients with the same tumor stage. The prognosis of NSCLC remains uncertain, mainly at the time of diagnosis (3). Genomic studies have shown that intra-tumor heterogeneity is a common feature of solid tumors, such as NSCLC (4, 5). However, because this procedure is invasive and sampling tissue is limited, it does not provide a complete characterization of tumor heterogeneity. Therefore, robust biomarkers that can provide a complete characterization of tumor heterogeneity may be of great value. Radiomics studies have shown promising results in revealing intra-tumor heterogeneity and predicting prognosis and treatment response in cancer patients (6–8). Radiomics is the high-throughput extraction of a large number of quantitative image features from medical images. It can capture information on the intensity, texture, and shape of lesions, which can be used to develop predictive models based on screened features to support personalized decision-making and treatment (9).

^18^F-FDG PET/CT has been used by clinicians to diagnose and evaluate NSCLC patients (8). Several studies have focused on tumor radiomic analysis of NSCLC patients to predict recurrence (10–12). The combination of radiomic features (RSs) and TNM staging or other clinicopathology (CP) data performs better than TNM staging alone in estimating disease-free survival in patients with early-stage NSCLC.

Recently, although radiomics has made significant progress in various malignancies (13–15), the radiomics literature for predicting prognosis in NSCLC is relatively sparse. A multimodality radiomics approach has great potential to address the limitations of unimodality models, as it can extract more meaningful features and thus provide a more robust description of the underlying tumor. In the present study, we retrospectively analyzed the data of NSCLC patients and screened feature parameters with predictive significance for prognosis by radiomics methods, aiming to construct a non-invasive deep learning model based on RSs of pretreatment PET/CT images to predict the survival prognosis of NSCLC patients.

## Materials and methods

### Patients

This retrospective study was approved by the institutional review board of the First Affiliated Hospital of Soochow University, and the informed consent was waived due to the retrospective nature of the study (ChiCTR2200062555).

NSCLC patients who underwent a combined imaging protocol of ^18^F-FDG PET/CT between January 2019 and June 2021 were recruited in our present retrospective study. Inclusion criteria were as follows: (a) patients who underwent biopsy or surgery of lung tumor; (b) patients with immunohistochemistry (IHC) examination of PD-L1 performed; (c) histological type and grade were pathologically proven; (d) standard ^18^F-FDG PET/CT was performed before biopsy or surgery; and (e) complete clinical profile. Exclusion criteria were as follows: (a) therapy (radiotherapy, chemotherapy, or chemoradiotherapy) was performed before ^18^F-FDG PET/CT and IHC; (b) patients with unknown histological grade; (c) the size of the primary lesion was too small for segmentation; (d) multi-center primary lung cancer; and (e) patients with other types of cancers or with incomplete clinical and imaging datasets. A total of 368 patients were enrolled in the present study, and they were randomly divided into the training (n = 257) and validation (n = 111) cohorts at a ratio of 7:3 (16).

### Acquisition and reconstruction of PET/CT images

The ^18^F-FDG PET/CT examination was performed after 6 h of fasting with blood glucose lower than 11.1 mmol/L. Approximately 40-60 min after the injection of ^18^F-FDG (4.07- 5.55 MBq/kg), PET/CT was performed from the base of the skull to the midthigh with 2-3 min per bed position (reconstructed by ordered subset expectation-maximization algorithm) using a Discovery PET/CT (General Electric Medical Systems, Milwaukee WI, USA) with low-dose CT parameters (140 kV, 120 mA, trans axial FOV of 70 cm, slice thickness 3.75 mm).

### Feature extraction and selection

The region of interest (ROI) of the lesion was outlined layer by layer on the image cross-section using LIFEx freeware (17) (v6.30 https://www.lifexsoft.org/) two experienced nuclear medicine physicians who were blinded to the clinical and pathological information of patients. Areas with abnormal uptake of ^18^F-FDG on PET and abnormal density on CT were defined as lesions. The volume of interest (VOI) in three-dimensional coordinates was automatically defined on PET images with the SUV_max_ threshold of 41%. The RSs were summarized across all VOIs. Spatial resampling had a voxel size of 2 × 2 × 2 mm. Intensity discretization for CT data was performed with the number of gray levels of 400 bins and absolute scale bounds from -1,000 and 3,000 HU, while it was conducted with 64 bins between 0 and 20 for PET data. The RSs were extracted from PET and CT images within the same VOI due to the excellent matching of PET and CT images. If the respiratory motion caused a mismatch between the PET and CT images, it could be adjusted manually. The RSs of PET and CT images were selected by the following procedure. Intra-class correlation coefficients (ICCs) were calculated to evaluate the repeatability of all the features, of which ICCs > 0.75 were selected for model construction (18, 19). Subsequently, the retained features were further selected by the least absolute shrinkage and selection operator (LASSO) regression algorithm. In addition, 10-fold cross-validation was applied to select the parameter of Lambda (l) to avoid overfitting.

### Patient follow-up

Patients were followed up from the first diagnosis of NSCLC by surgery or puncture pathology to June 30, 2021 (the last follow-up date), in conjunction with outpatient review data or by telephone. Progression-free survival (PFS) was defined as the time from the first diagnosis of NSCLC by surgery or puncture pathology to the date of tumor progression, death, or the follow-up cut-off date. Overall survival (OS) was defined as the time from the first diagnosis of NSCLC by surgery or puncture pathology to the date of death or the follow-up cut-off date. Receiver operating characteristic (ROC) curves and Harrell’s consistency index (C-index) were used to assess the model’s capability to predict outcomes in both training and validation cohorts. Moreover, calibration curves were produced to assess the model’s calibration. In order to evaluate the clinical value of various models, decision curve analysis (DCA) was employed to calculate the net benefit at various threshold probabilities.

### Statistical analysis

IBM SPSS statistics version 26.0, Python version 3.0 (https://www.python.org), MedCalc software (MedCalc Software, Ostend, Belgium), and R 3.6.3 software package were used for statistical analyses. P < 0.05 was considered statistically significant. The continuous variables were transformed into dichotomous variables for further statistical analysis. The Kaplan⁃Meier (KM) curve was used for survival analysis, the log⁃rank test was used for univariate survival analysis, Cox proportional risk regression model was used for the multifactor prognostic analysis of significant influencing factors, and the hazard ratio (HR) and its 95% CI were estimated.

## Results

### Patient characteristics

A total of 368 patients were enrolled in this study. **Table 1** summarizes the clinical characteristics of patients in the training and validation cohorts. Of these patients, there were not any statistically significant differences in the clinical characteristics between the training and validation cohorts. Of the 368 patients, 20.92% (77/368) had disease progression, and 4.62% (17/368) died. Of the 257 patients in the training group, 21.4% (55/257) had disease progression, and 3.5% (9/257) died, with a median OS and PFS of 16 and 14 months, respectively. Of the 111 patients in the validation group, 19.82% (22/111) had disease progression, 5.41% (6/111) died, and the median OS and PFS were 17 and 14 months, respectively.

**Table 1:** Clinical characteristics of patients in the training and validation cohorts.

Characteristics	Total (n=368)	Training (n=257)	Validation (n=111)	t/χ^2^	*P*
Sex				3.808	0.051
Male	238	158	80		
Female	130	99	31		
Age, median ± SD, years		64.10 ± 9.28	65.82 ± 8.83	1.691	0.092
Tumor location				0.993	0.319
Left lung	210	151	59		
Right lung	158	106	52		
Histologic type, No. (%)				3.047	0.081
Squamous cell carcinoma	100	63	37		
Adenocarcinoma	268	194	74		
LN				0.504	0.478
Positive	172	117	55		
Negative	196	140	56		
Metastasis				0.931	0.334
Positive	78	51	27		
Negative	290	206	84		
TNM stage, No. (%)				0.033	0.856
I-II	183	127	56		
III- IV	185	130	55		
Smoking history				0.949	0.33
Smoker	198	134	64		
Never	170	123	47		
Ki-67				0.098	0.754
<20%	102	70	32		
≥20%	266	187	79		
Treatment				0.025	0.874
Non targeted therapy	138	95	42		
Targeted therapy	231	162	69		
PD-L1 status				0.023	0.878
<1%	147	102	45		
≥1%	221	155	66		
PD-L1 status				0.817	0.366
<50%	288	206	82		
≥50%	77	51	26		

LN, lymph node; PD-L1, programmed cell death-Ligand 1; **χ^2^**, chi-square test; P-value of the last column show differences of variables in the training set and testing set.

### RS selection and R-signature building

**Figure 1:**
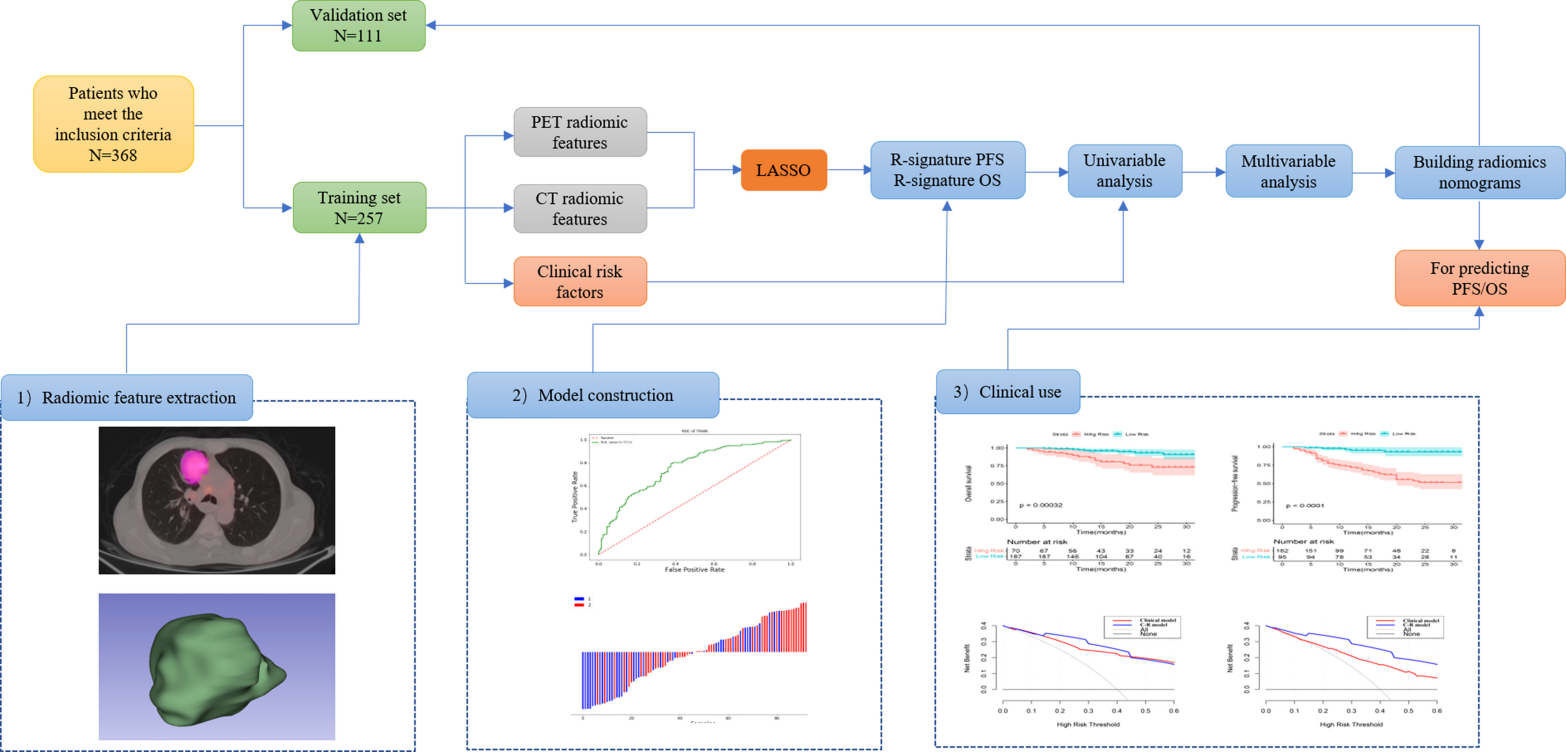
Workflow of the radiomics analysis. 1) tumor masking and feature extraction; 2) model construction; 3) clinical use.

Prognosis-related features were selected from the LASSO regression in the training cohort. Three PET RSs (DISCRETIZED_HISTO_Entropy_log2, CONVENTIONAL_SUVbwmin, and CONVENTIONAL_SUVbwmax) and two CT RSs (NGLDM_Contrast and GLZLM_SZLGE) were selected for predicting PFS. Three PET RSs (DISCRETIZED_SUVbwmin, GLRLM_RLNU, and GLRLM_RP) and three CT RSs (CONVENTIONAL_Humax, GLCM_Entropy_log10, and GLZLM_ZLNU) were selected for predicting OS. Rad-score (PFS) = DISCRETIZED_HISTO_Entropy_log2 × 0.3397+CONVENTIONAL_SUVbwmin × 0.1288 - GLRLM_SRHGE × 0.3009 -NGLDM_Contrast × 0.3187 + GLZLM_SZLGE × 0.2147. Rad-score (OS) = CONVENTIONAL_Humax × 0.7139 - GLCM_Entropy_log10 × 0.8292 + GLZLM_ZLNU × 0.3407 + DISCRETIZED_SUVbwmin × 0.2364 + GLRLM_RLNU × 0.6754 - GLRLM_RP × 1.4249. Besides, the Rad-score was calculated for predicting PFS (AUC = 0.739) and OS (AUC = 0.696) based on the above-selected RSs. The optimal cut-off values of the Rad-score generated by the ROC analysis were 0.375 for PFS and 0.508 for OS in the training cohort. Accordingly, patients were classified into the low-risk and high-risk groups. The radiomic workflow is presented in **Figure 1**.

### Cox analysis of prognostic influences

**Figure 2:**
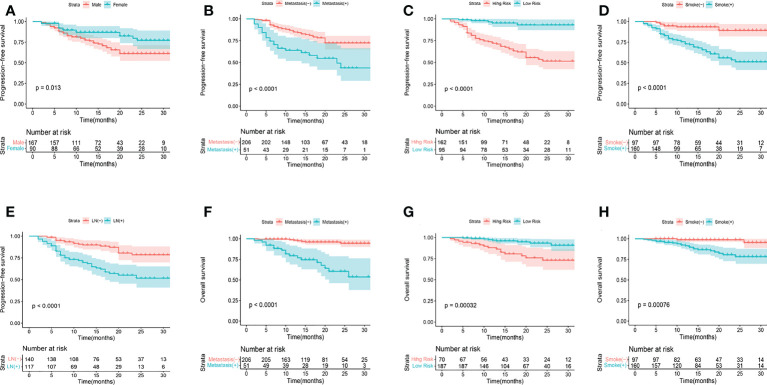
KM analyses for PFS **(A-E)** and OS **(F-H)** according to gender, metastasis, smoking history, R-signatures, and lymph node metastasis in the training cohort.

**Figure 3:**
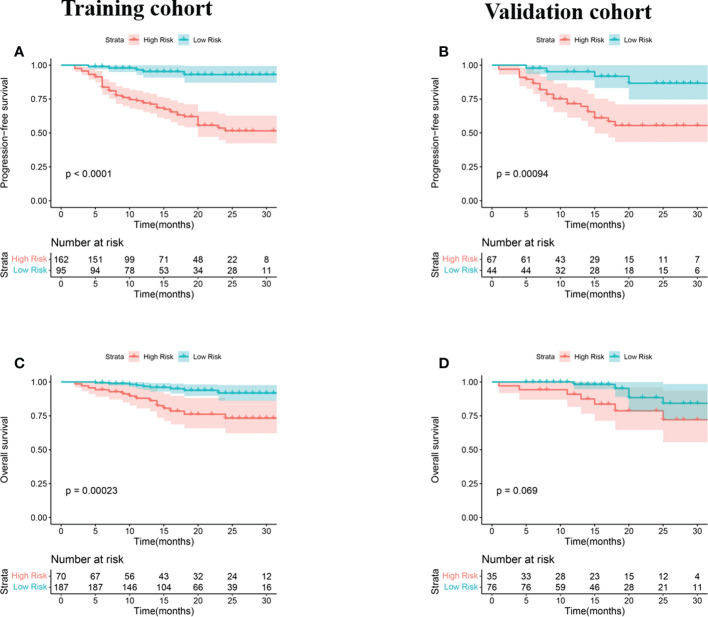
KM analyses for patients stratified by risk classification according to R-signatures for patients in the training **(A, C)** and validation **(B, D)** cohorts.

**Figure 4:**
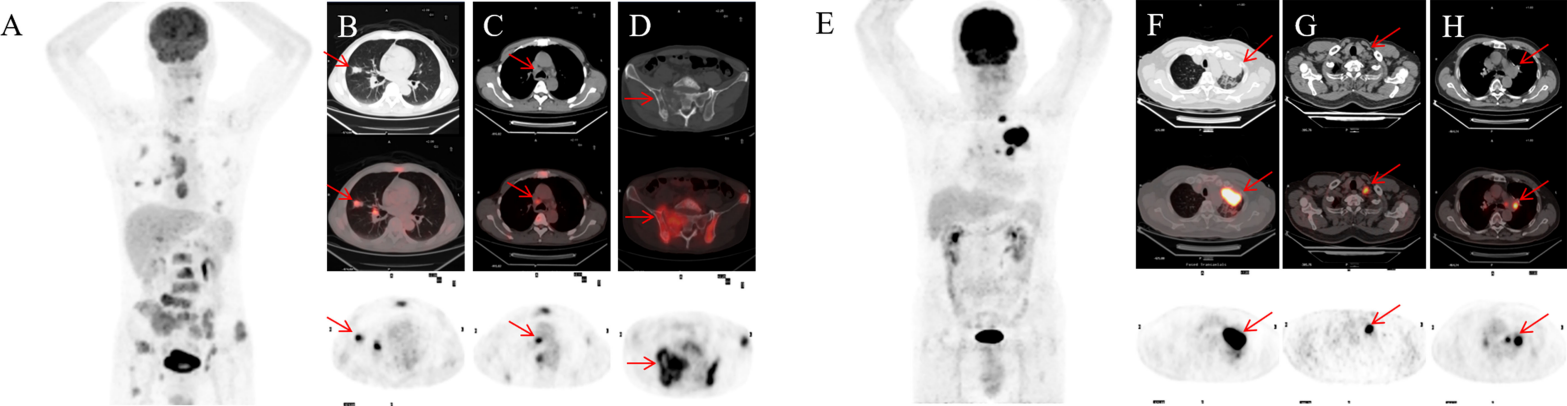
A 55-year-old male NSCLC patient with a history of smoking **(A)**. Before treatment, 18F-FDG PET-CT revealed lesions in the right upper lobe and lymph node and pelvic bone metastases **(B-D)**. After 4 months of targeted therapy, the patient died. A 67-year-old male NSCLC patient with a smoking history **(E)**. Before treatment, 18F-FDG PET-CT found lesions in the upper lobe of the left lung, mediastinal and hilar lymph node metastasis **(F-H)**. At 11 months after targeted therapy, the patient’s disease progresses.

Univariate and multivariate Cox regression analyses were used to determine the relationship between clinical features and imaging indicators with survival (**Table 2** and **Table 3**; **Figure 2**). Using PFS as the study endpoint, KM univariate analysis showed that gender, TNM staging, pathology, smoking history, treatment modality, distant metastasis, Ki-67, lymph node metastasis, SUV_max_, MTV, TLG, and Rad-score were factors influencing the PFS of patients (all P < 0.05). Cox multifactorial analysis showed that gender, smoking history, lymph node metastasis, distant metastasis, and Rad-score were independent prognostic factors affecting PFS (all *P* < 0.05). With OS as the study endpoint, KM univariate analysis showed that gender, PD-L1 50%, smoking history, distant metastasis, lymph node metastasis, SUV_max_, MTV, TLG, and Rad-score were factors influencing the OS of patients (all P < 0.05). Cox multivariate analysis showed that smoking history, distant metastasis, and Rad-score were factors influencing the OS of patients (all P < 0.05). Furthermore, Rad-score and clinical factors were significantly correlated with PFS and OS in the training cohort and validation cohort (**Figure 3** and **Figure 4**).

**Table 2:** The results of the univariate Cox regression analysis.

Variable	PFS		OS	
	HR (95%CI)	*P*	HR (95%CI)	*P*
Gender	1.993 (1.199-3.311)	0.029*	6.79 (2.118-21.76)	0.033*
PD-L1 1%	1.034 (0.625-1.709)	0.896	1.41 (0.447-4.445)	0.550
PD-L1 50%	0.610 (0.322-1.153)	0.075	0.327 (0.077-1.387)	0.045*
TNM	0.460 (0.280-0.756)	0.002*	0.585 (0.188-1.824)	0.353
Pathology	1.765 (1.004-3.102)	0.025*	0.950 (0.262-3.454)	0.939
Smoke	0.188 (0.114-0.307)	<0.001*	0.127 (0.056-0.285)	0.001*
Treatment	2.526 (1.503-4.245)	0.001*	2.619 (0.799-8.589)	0.087
Metastasis	0.391 (0.204-0.751)	<0.001*	0.326 (0.077-1.384)	0.044*
Ki-67	0.265 (0.156-0.449)	0.001*	0.470 (0.137-1.618)	0.317
LN	0.310 (0.188-0.511)	<0.001*	0.189 (0.084-0.426)	<0.001*
SUV_max_	5.428 (3.196-9.222)	<0.001*	4.252 (1.243-14.54)	0.021*
MTV	2.706 (1.632-4.489)	0.001*	4.446 (1.403-14.09)	0.014*
TLG (mL)	4.938 (2.993-8.147)	<0.001*	3.159 (0.903-11.06)	0.007*
Rad-score	8.178 (4.953-13.50)	<0.001*	27.61 (7.883-96.72)	<0.001*

LN, lymph node; SUV_max_, standardized uptake value max; MTV, total metabolic tumor volume; TLG, total lesion glycolysis.

*P < 0.05.

**Table 3:** The results of the multivariate Cox regression analysis.

Variable	PFS		OS	
	HR (95%CI)	*P*	HR (95%CI)	*P*
Gender	2.394 (1.086-5.276)	0.030*	NS	
PD-L1 50%	NA	NA	NS	
TNM	NS		NA	NA
Pathology	NS		NA	NA
Smoke	4.989 (2.098-11.860)	<0.001*	5.507 (1.280-23.697)	0.022*
Treatment	NS		NA	NA
Metastasis	1.949 (1.127-3.371)	0.017*	8.785 (3.632-21.248)	<0.001*
Ki-67	NS		NA	NA
LN	2.185 (0.984-4.855)	0.045	NS	
SUV_max_	NS		NS	
MTV	NS		NS	
TLG (mL)	NS		NS	
Rad-score	0.1952 (0.045-0.855)	0.030*	0.344(0.150-0.792)	0.012*

HR, hazard ratio; LN lymph node; SUV_max_, standardized uptake value max; MTV, total metabolic tumor volume; TLG, total lesion glycolysis.

NS, not significant; NA, not apply.

*P < 0.05.

### Assessment and validation of the models for predicting PFS and OS

**Figure 5:**
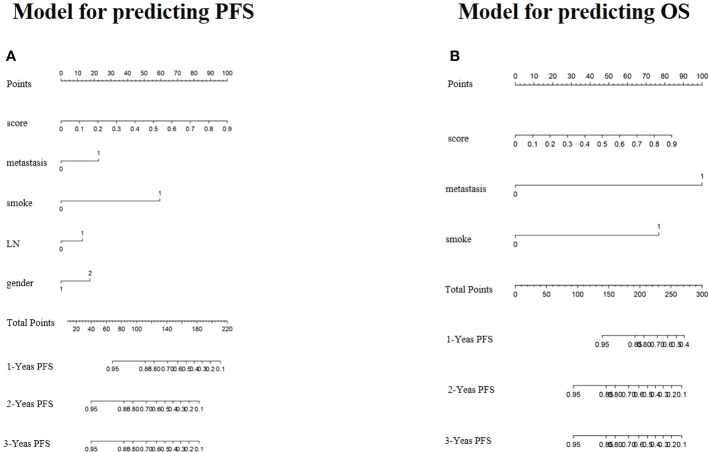
Radiomics nomogram for predicting PFS **(A)** and OS **(B)** of NSCLC.

To provide a quantitative method to predict patients’ probability of 1-, 2-, and 3-year PFS and OS, a radiomics nomogram combining clinical factors was established (**Figure 5**).

For the training cohort, the C-index and AUC of the C-R model were 0.775 (95% CI: 0.722-0.828) and 0.780 (95% CI: 0.724-0.829) for PFS prediction, respectively. These values were superior to those of the clinical model (C-index: 0.748, 95% CI: 0.686-0.810, and AUC: 0.758, 95% CI: 0.701-0.809). For OS prediction, the C-index and AUC of the C-R model were 0.881 (95% CI: 0.829-0.933) and 0.891 (95% CI: 0.846-0.926), respectively, and these values were superior to those of the clinical model (C-index: 0.834, 95% CI: 0.768-0.899, and AUC: 0.846, 95% CI: 0.796-0.888) (**Table 4**).

**Table 4:** The Harrell’s C-index and AUC results in the training and validation cohorts.

	Training cohort		Validation cohort	
	C-index (95% CI)	AUC (95% CI)	C-index (95% CI)	AUC (95% CI)
PFS				
Clinical model	0.748 (0.686-0.810)	0.758 (0.701-0.809)	0.729 (0.639-0.819)	0.776 (0.687-0.850)
C-R model	0.775 (0.722-0.828)	0.780 (0.724-0.829)	0.755 (0.678-0.831)	0.791 (0.704-0.863)
OS				
Clinical model	0.834 (0.768-0.899)	0.846 (0.796-0.888)	0.832 (0.724-0.939)	0.850 (0.769-0.910)
C-R model	0.881 (0.829-0.933)	0.891 (0.846-0.926)	0.867 (0.781-0.953)	0.874 (0.797-0.929)

CI, confidence interval; AUC, area under the curve.

For the validation cohort, the C-index of the two models was 0.729 (95% CI: 0.639-0.819) and 0.755 (95% CI: 0.678-0.831) for predicting PFS, and 0.832 (95% CI: 0.724-0.939) and 0.867 (95% CI: 0.781-0.953) for predicting OS. Using ROC curve analysis, we found that the C-R model had a higher AUC compared with the clinical model for predicting PFS (AUC = 0.791 *VS.* 0.776) and OS (AUC = 0.874 *VS.* 0.850) (**Table 4**).

### Clinical use

**Figure 6:**
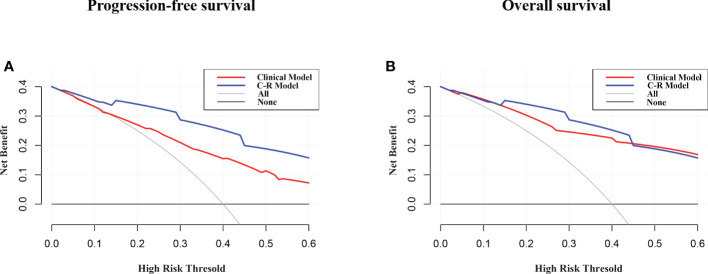
DCA of PFS **(A)** and OS **(B)** for the model. The y-axis measures the net benefit. The net benefit was calculated by summing the benefits (true positive results) and subtracting the harms (false-positive results), weighting the latter by a factor related to the relative harm of undetected cancer compared with the harm of unnecessary treatment. The C-R model had the highest net benefit compared with the clinical model.

**Figure 6** presents the DCA for the radiomics model and clinical model. DCA indicated that the net benefit of the radiomics nomogram was faintly higher compared with the clinical nomogram for both PFS and OS.

## Discussion

Currently, lung cancer remains the leading cause of cancer-related deaths worldwide (1). The survival of NSCLC patients varies greatly among individuals, and the survival prognosis is affected by many factors. Our study found that Rad-scores on ^18^F-FDG PET/CT in cox regression analysis were independent predictors of PFS and OS. For better application in clinical practice, we constructed a clinical model for predicting disease progression and a complex model combining clinical factors and Rad-scores. The complex model showed superior predictive performance in both training and validation cohorts compared with the clinical model. In the training group, the C⁃index of the clinical model for predicting PFS and OS was 0.748 and 0.834, respectively, and the C⁃index of the complex model for predicting PFS and OS was increased to 0.775 and 0.881 after the addition of imaging histological features, respectively, suggesting that the complex model was the best model for predicting survival prognosis of NSCLC.

Traditional prognostic assessment methods, such as TNM staging, are important in guiding the formulation of rational treatment plans and prognosis assessments for lung cancer patients (20–22). However, even at the same TNM stage, the individual differences between patients make the prognosis significantly different, and the differences are magnified as the stage increases. Therefore, it is difficult to accurately assess the survival of NSCLC patients based on the TNM staging system alone. ^18^F-FDG PET/CT is a whole-body imaging examination that is widely used for the staging of malignant tumors. Previous studies have found that tumor FDG uptake can be used as a prognostic marker in patients with advanced and inoperable early-stage NSCLC (23, 24). Meta-analysis of IASLC shows that patients with higher preoperative primary tumor SUV_max_ have lower survival rates and shorter survival times (25). Konings R et al. have shown that the primary tumor SUV_max_ has a predictive value for the prognosis of NSCLC (26, 27). As the SUV-based approach is influenced by many physiological and technical factors, such as patient preparation, coordination of image acquisition, reconstruction, and analysis (28, 29), other features derived from PET/CT images are currently being explored.

Radiomics is the comprehensive quantification of tumor phenotypes through the application of numerous quantitative imaging features, which may reflect changes in human tissues at the cellular and genetic levels and offer more thorough details on tumor biology and the microenvironment in addition to visual features (30, 31). Radiomics has been used for the diagnosis, response assessment, and survival prognosis of various cancers (32–34). Currently, there are few studies on PET/CT imaging histology to predict the prognosis of NSCLC. It has been reported that imaging features are an independent prognostic factor for NSCLC and are associated with multiple clinical endpoints (35, 36). Therefore, we attempted to establish a complex model to assess the potential prognostic value of NSCLC patients by combining PET/CT-based radiomics features with clinical features.

Our study investigated the potential prognostic value of imaging histological features derived from pretreatment ^18^F-FDG PET/CT images in NSCLC patients. In this study, a complex model combining radiomics and clinical factors was found to be more effective in predicting PFS and OS, improving the efficacy of traditional clinical parameters in assessing the prognosis of NSCLC and aiding the prognosis of NSCLC patients. These findings were consistent with previous studies showing that RSs extracted from PET/CT images have potential implications for assessing disease status and risk stratification in NSCLC (37, 38).

The present study has a number of limitations. Firstly, although the patients included in this study were the largest patient group compared with previous studies, the sample size was relatively small, and it was a retrospective study. Secondly, the model was developed and validated based on a single institution dataset. The relationship between clinicopathological parameters and prognosis might have been influenced by the sample size. Future prospective multi-center studies with large data are required to obtain more accurate and reproducible imaging histological features. Finally, the effectiveness of adjuvant therapy for patients with high Rad-scores according to ^18^F-FDG PET/CT nomograms should also be evaluated in the future.

## Conclusions

In conclusion, a complex model combining clinicopathological factors and radiomic features extracted from ^18^F-FDG PET/CT images had the potential to predict PFS and OS, and the nomogram analyses could provide more accurate individualized predictions of PFS and OS for patients, which in turn had the potential to help clinicians make more informed decisions in clinical practice.

## Data availability statement

The raw data supporting the conclusions of this article will be made available by the authors, without undue reservation.

## Ethics statement

This retrospective study was approved by the institutional review board of the First Affiliated Hospital of Soochow University, and the informed consent was waived due to the retrospective nature of the study (ChiCTR2200062555). The ethics committee waived the requirement of written informed consent for participation.

## Author contributions

JL: data curation and writing- original draft preparation. BZ: conceptualization, methodology, and software. SG: visualization and investigation. SD: supervision. CH: software and validation. SS: writing- reviewing and editing. All authors contributed to the article and approved the submitted version.

## Funding

This research was funded by the National Natural Science Foundation of China (No. 81601522), Medical Youth Talent Project of Jiangsu Province (No. QNRC2016749), Gusu Health Talent Program (No. GSWS2020013), Suzhou People’s Livelihood Science and Technology Project (No. SYS2019038), Project of State Key Laboratory of Radiation Medicine and Protection, Soochow University (No. GZK1202127), and the Open Foundation of Nuclear Medicine Laboratory of Mianyang Central Hospital, (No. 2021HYX023 and 2021HYX029).

## Conflict of interest

The authors declare that the research was conducted in the absence of any commercial or financial relationships that could be construed as a potential conflict of interest.

## Publisher’s note

All claims expressed in this article are solely those of the authors and do not necessarily represent those of their affiliated organizations, or those of the publisher, the editors and the reviewers. Any product that may be evaluated in this article, or claim that may be made by its manufacturer, is not guaranteed or endorsed by the publisher.
